# Follicular Becker's Nevus: A Case Report of an Uncommon Clinical Variant

**DOI:** 10.7759/cureus.58264

**Published:** 2024-04-14

**Authors:** Anannya S, Afthab Jameela Wahab, Sheba Mariam Jacob, Vimal Chander R

**Affiliations:** 1 Department of Dermatology, Saveetha Medical College, Chennai, IND; 2 Department of Pathology, Saveetha Medical College, Chennai, IND

**Keywords:** macular lesions, perifollicular, dermoscopy, hypopigmentation, becker’s nevus

## Abstract

Pigmented hairy epidermal nevus, also known as Becker's nevus, has a typical description as a unilateral, hairy in appearance, light to dark brown patch with an irregular but clearly defined border. However, the exact aetiopathogenesis is still poorly comprehended. We report the case of a 19-year-old female who presented with asymptomatic brownish-pigmented macular lesions on the right breast that had slowly increased in size over the past three years. Upon cutaneous inspection, the right breast had 3-5 mm rounded and oval perifollicular macules that ranged from light to dark brown hue without increased hair growth. The macules were discrete and in no particular pattern. Dermoscopy of the lesions showed well-defined perifollicular hypopigmentation surrounded by a pigmented network-like pattern. Histopathology of a punch biopsy taken from one of the follicular lesions demonstrated an increase in basal layer pigmentation with elongation of rete ridges and acanthosis, consistent with Becker's nevus. The patient underwent three sittings of diode laser therapy, once in four weeks, with slight improvement in pigmentation.

## Introduction

Samuel William Becker originally described Becker's nevus in 1949, otherwise called as pigmented hairy epidermal nevus, as an acquired condition characterised by localised hypermelanosis and hypertrichosis [1]. The shoulders, the anterior chest, and sometimes the scapula may be involved where the lesion is usually a hyperpigmented patch with sharply outlined borders. In rare instances, it can be found on the lower back and lower limbs [2,3]. It mostly occurs around puberty but can present at birth or manifest in early childhood [4]. Studies have reported familial cases [5], its association with other developmental anomalies, or the absence of hypertrichosis [6-9]. It has also been documented that Becker's nevus can at times develop in tandem with unilateral breast hypoplasia or additional cutaneous and musculoskeletal anomalies and this may be referred to as "Becker's nevus syndrome" [10]. Histopathological examination shows epidermis with mild acanthosis, hyperkeratosis, and regular elongation of rete ridges. The melanocyte number is constant or slightly elevated, but the quantity of melanin is considerably high. The papillary dermis shows the presence of melanophages.

To the best of our knowledge, only 11 cases of Becker's nevus solely manifesting as follicular lesions have been reported in the literature since its first description 73 years ago. Our patient was a 19-year-old female with 3-5 mm rounded and oval perifollicular macules that ranged from light to dark brown hue with no hypertrichosis. Clinicopathological correlation was used to establish the diagnosis. Diode laser was tried as a therapeutic modality in this case. The aetiopathogenesis of Becker's nevus is still being debated, and this atypical variant may suggest that follicular epithelium holds a significant role in it.

## Case presentation

**Figure 1:**
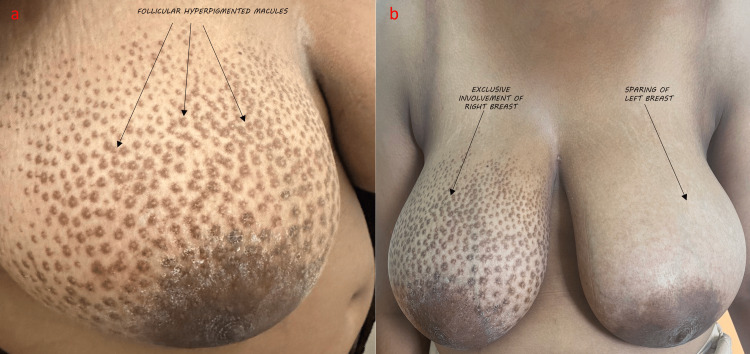
a) Numerous, hyperpigmented, follicular macules are seen on all four quadrants of the right breast. b) Exclusive involvement of the right breast with hyperpigmented macules in all four quadrants and relative sparing of the left breast

A 19-year-old female presented with asymptomatic brownish-pigmented macular lesions on the right breast that had been slowly increasing in size over the past three years. Cutaneous examination revealed light to dark brown-colored round to oval 3-5 mm perifollicular macules on all four quadrants of the right breast without increased hair growth (Figure 1). The macules were discrete and in no particular pattern. The right nipple seemed normal and no apparent skeletal deformity was noted in the vicinity. The left breast was normal on examination. There were no skin lesions elsewhere on the body or any mucosal involvement.

**Figure 2:**
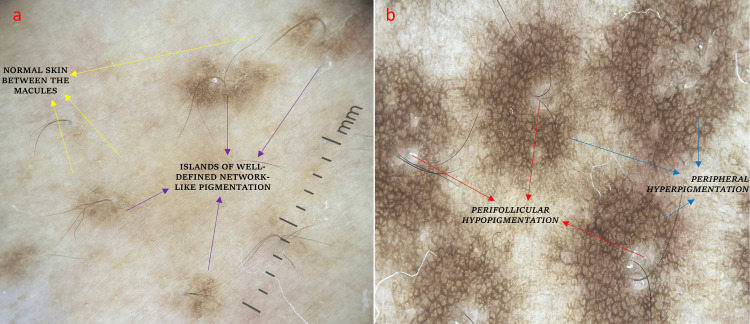
Dermoscopy (DermLite DL4) findings are a) islands of well-defined dark pigment network (violet arrows), interspersed between these islands are areas of normal skin with a normal skin pigment network (yellow arrows), and b) each individual follicle shows immediate perifollicular hypopigmentation (red arrows) surrounded by a hyperpigmented zone (blue arrows), which corresponds clinically to hyperpigmented macules representing follicular variant of Becker's nevus

Comprehensive clinical evaluation ruled out the involvement of other systems. Ultrasonography of the breasts showed no parenchymal abnormalities, and biochemical and hormonal profiles were found to be normal. Dermoscopy of the breast lesions showed well-defined perifollicular hypopigmentation surrounded by a dark pigment network (Figure 2).

**Figure 3:**
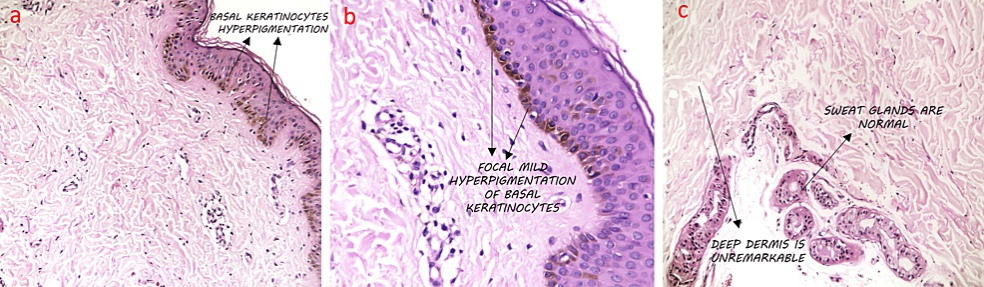
Histopathological features are a (H&E ×100) and b (H&E ×400): skin with epidermis showing focal mild hyperpigmentation of basal keratinocytes with the superficial dermis showing minimal perivascular lymphoplasmacytic infiltrates. c) (H&E ×400) The deep dermis and sweat glands appearing unremarkable

Histopathology of a punch biopsy taken from one of the follicular lesions revealed a darkly pigmented basal cell layer with regular elongation of rete ridges and mild acanthosis, and these features are consistent with Becker's nevus (Figure 3).

The patient underwent three sittings of diode laser treatment once in four weeks without much improvement in pigmentation.

## Discussion

Becker’s nevus is a disorder of epidermal and dermal interaction due to hormonal influences. The aetiopathogenesis remains unclear; many theories have been proposed explaining the role of hormonal influence through androgen receptors [11] and genetic factors. However, all of the characteristics linked to Becker's nevus cannot be fully explained by any of the numerous theories that have been put forth.

Manchanda et al. presented a case of Becker's nevus with predominantly follicular involvement [12]. Dermoscopy showed a continuous sheet of pigmentation and islands of pigmentation with the network of lines having uniform color and thickness across the lesion. In our case, dermoscopy of the macular lesions showed well-defined perifollicular hypopigmentation surrounded by a dark pigment network.

Manchanda et al. explained in the clinical presentation from four patients with follicular lesions that the possibility of follicular epithelium contributing to the aetiopathogenesis of Becker's nevus warranted more research [12].

Manchanda et al. in one case reported asymptomatic brownish-pigmented macules on the anterior right shoulder that had increased slowly over a year, without associated skeletal defect. Our patient also showed asymptomatic brownish-pigmented macular lesions on the right breast that increased in extent over three years without any skeletal defect, while in another male patient, Manchada et al. noted multiple grouped asymptomatic dark brown to blackish-colored perifollicular macules and papules measuring less than 4 mm on the right upper back and right upper extremity present for a year with hypertrichosis, without associated structural abnormality [12].

Zhang et al. reported a female with multiple asymptomatic grouped brown macules on the medial aspect of the left upper arm for 12 years. The pigmented lesions gradually increased in the initial phase and then stabilized, with no systemic abnormality. Dermoscopy showed circular brown perifollicular macules with thick hairs in the centre. The histopathological examination showed hyperkeratosis, acanthosis, and basal layer hyperpigmentation [13].

Ranglani and Malakar reported an 18-year-old male with asymptomatic, numerous, well-defined, folliculocentric hyperpigmented macules over the right side of the upper chest and supraclavicular region tending to coalesce at one side, with terminal hair located at the centre of each macule since 18 months [14].

Pangti et al. presented a 50-year-old male with several asymptomatic, 3-mm-sized, follicular, hyperpigmented macules occupying a 7 cm×5 cm area across the left chest that had been present since adolescence. No skeletal defects, acneiform eruption, or hypertrichosis was present. There were mild acanthosis and papillomatosis in the epidermis with considerable basal cell melanization upon histological inspection. Broad, interconnecting rete ridges were occasionally seen, supporting the diagnosis of follicular Becker's nevus [15].

On examination, our patient showed pale to deep brown-colored, rounded and oval in form, 3-5 mm perifollicular macules on all four quadrants of the right breast without increased hair growth. Histopathology of a punch biopsy taken from one follicular lesion demonstrated increased basal layer melanization with regularly elongated rete ridges and acanthosis, consistent with Becker's nevus.

A variety of lasers are used either singly or in combination, with wavelengths between 504 and 10,600 nm, to treat the hyperpigmentation and hypertrichosis in Becker's nevus [16]. Our patient underwent three sittings of diode laser therapy once in four weeks without much improvement in pigmentation.

## Conclusions

The aetiopathogenesis of Becker's nevus is yet to be fully understood. However, this follicular Becker's nevus atypical variant may suggest that follicular epithelium can hold a significant role in its aetiopathogenesis. This case is being presented for its rarity and to reiterate the need to further evaluate the role of the follicular epithelium in causing Becker's nevus.
